# Supplementary material for: High-Throughput Sequencing Reveals New Viroid Species in Opuntia in Mexico
Source: Viruses. 2024 Jul 23;16(8):1177. doi: 10.3390/v16081177 (PMC11359548; doi:10.3390/v16081177)
Supplement: Supplementary file 1 [file viruses-16-01177-s001.zip › Suplementary Figure S1-9.pdf]

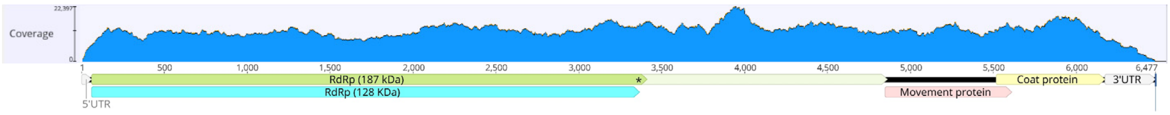

Figure S1. Read coverage over the genome of Opuntia virus 2, isolate EM_T2, obtained from nopalitos. Open reading frame (ORF) organization and annotation are shown in different colors. *Indicates the site of the readthrough leaky termination codon of the 128-kDa protein.

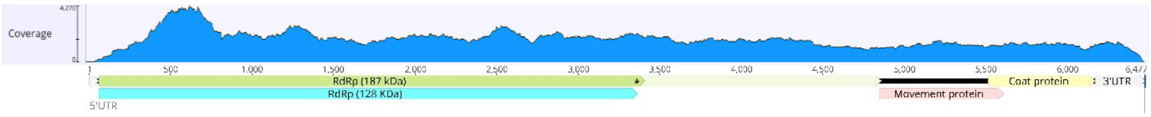

Figure S2. Read coverage over the genome of Opuntia virus 2, isolate EM_T1, obtained from cactus pears. Open reading frame (ORF) organization and annotation are shown in different colors. *Indicates the site of the readthrough leaky termination codon of the 128-kDa protein.

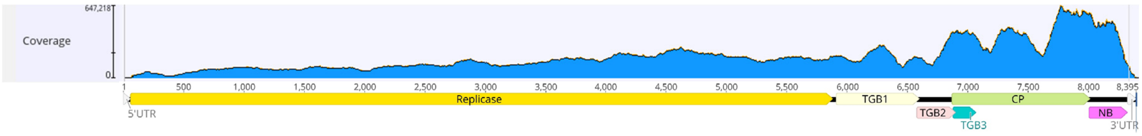

Figure S3. Read coverage over the genome of Cactus carlavirus 1, isolate EM_C2, obtained from nopalitos. Open reading frame (ORF) organization and annotation are shown in different colors. TGB, triple gene block; CP, capsid protein; NB, nucleic acid binding protein.

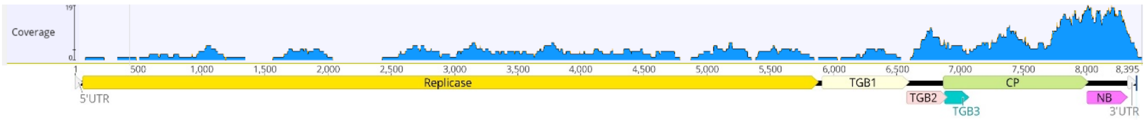

Figure S4. Read coverage over the genome of Cactus carlavirus 1, isolate EM_C1, obtained from cactus pears. Open reading frame (ORF) organization and annotation are shown in different colors. TGB, triple gene block; CP, capsid protein; NB, nucleic acid binding protein.

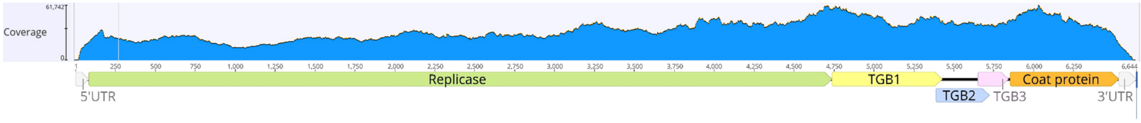

Figure S5. Read coverage over the genome of Opuntia potexvirus A, isolate EM_A2, obtained from nopalitos. Open reading frame (ORF) organization and annotation are shown in different colors. TGB, triple gene block.

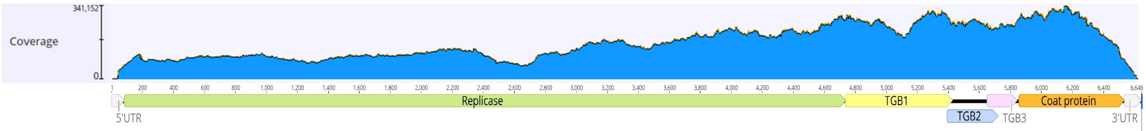

Figure S6. Read coverage over the genome of Opuntia potexvirus A, isolate EM_A1, obtained from cactus pears. Open reading frame (ORF) organization and annotation are shown in different colors. TGB, triple gene block.

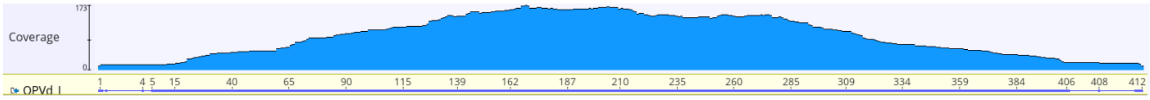

Figure S7. Read coverage over the genome of Opuntia viroid 1, isolate EDMEX-V1, from nopalitos.

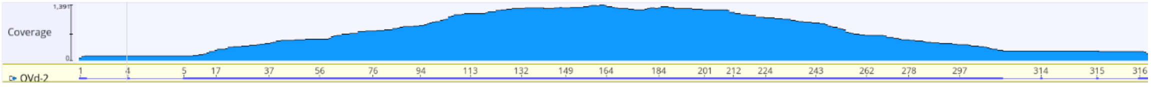

Figure S8. Read coverage over the genome of Opuntia viroid 2, isolate EDMEX-T1, from nopalitos.

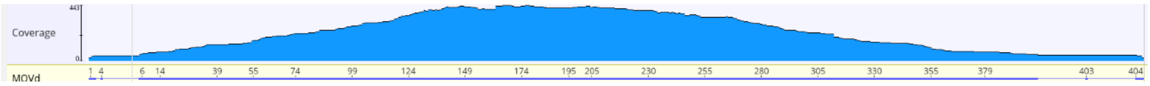

Figure S9. Read coverage over the genome of Mexican opuntia viroid, isolate V1, from nopalitos.
